# Effects of moxibustion on pain behaviors in patients with rheumatoid arthritis: A meta-analysis

**DOI:** 10.1097/MD.0000000000016413

**Published:** 2019-07-26

**Authors:** Biyu Shen, Qian Sun, Haoyang Chen, Yongchang Li, Xian Du, Huiling Li, Guang-yin Xu

**Affiliations:** a Center for Translational Pain Medicine, Institute of Neuroscience, Soochow University; b Nursing School of Soochow University, Suzhou; c Department of Nursing, The Second Affiliated Hospital of Nantong University; d Nursing School of Nantong University, Nantong, China.

**Keywords:** meta-analysis, moxibustion, pain, rheumatoid arthritis

## Abstract

**Background:**

Pain is the main symptom of patients with rheumatoid arthritis (RA). Reports of the effects of moxibustion on patients with rheumatoid arthritis have reached various conclusions. The aim of this meta-analysis was to evaluate the effect of moxibustion on pain in patients with RA.

**Methods:**

A systematic search of MEDLINE, EMBASE, the Cochrane Library, and the Chinese databases Wan Fang Med Database, CNKI, and VIP (until November, 2018) was used to identify studies reporting pain (on a visual analogue scale (VAS)), erythrocyte sedimentation rate (ESR), C-reactive protein (CRP) and rheumatoid factor (RF) levels, response rate, and the ACR50 rate in patients with RA. Results were expressed as mean difference (MD) and 95% confidence intervals (CI).

**Results:**

Six studies involving 281 participants were included. Moxibustion had significant effects on pain (VAS: MD = −0.53, 95% CI [−0.94, −0.12], *P* =.01). Moreover, moxibustion had effects on CRP (MD = −2.84, 95% CI [−5.13, −0.55], *P* =.01), ESR (MD = −8.44, 95% CI ([−13.19, −3.68], *P* =.0005), and RF (MD = −6.39, 95% CI [−18.57, 5.79], *P* =.30). Additionally, it had effects on response rate (n = 249, RR = 1.26, 95% CI [1.11, 1.43], *P* =.0004) and ACR50 rate (n = 140, RR = 1.44, 95% CI [1.11, 1.88], *P* =.007).

**Conclusion:**

We found that moxibustion with Western medicine therapy is superior to Western medicine therapy alone for pain in patients with RA. Moxibustion had significant effects on pain in patients with RA, but the effects of moxibustion on inflammatory factors in RA were unclear.

## 1 Introduction

Rheumatoid arthritis (RA) is a chronic autoimmune disease and is the commonest inflammatory joint disease. Its global prevalence ranges from 0.2% to 1.2%, and it affects 0.28% to 0.45% of Chinese individuals. Women are 3 times more frequently affected than men.^[1]^

RA is characterized by pain, swelling and stiffness of the joints, fatigue, and consequently reduced quality of life. In the long term, it causes irreversible joint destruction, deformities, visceral manifestations, and other diseases.^[2]^ Thus, RA has severe physical and mental effects on patients and their family members.^[3]^ Additionally, increasing evidence shows that pain cognition has important roles in health-care cost and disability rate.

Moxibustion is a traditional Chinese therapy. It uses the heat generated by burning herbal preparations containing mugwort (*Artemisia vulgaris*) to stimulate acupuncture points. Mugwort is used in the traditional Chinese medicine (TCM) in a pulverized and aged form called moxa. There are 2 types of moxibustion. Direct moxibustion involves direct application of moxa to the skin surface at the acupuncture point. It has further subdivisions, including scarring moxibustion (burning moxa on the skin), warming moxibustion (burning moxa above the skin). In indirect moxibustion, some insulating materials (ginger, salts, etc.) are placed between the moxa cone and the skin.^[4,5]^ Previous systematic reviews of moxibustion for RA have shown that moxibustion might be beneficial to RA patients.^[6,7]^ One^[6]^ of these studies included 8 randomized clinical trials (RCTs) in which moxibustion was compared with drug therapy in RA patients, and suggested that moxibustion might be beneficial. The second review^[7]^ also described some favorable effects of moxibustion in the treatment for RA.

Pain is often considered a surrogate marker for inflammatory disease activity in RA.^[8]^ It is the single largest determinant of global disease activity in patient assessments. Pain coping, assessed in the early stage of the disease, can affect long-term functional disability and pain in RA, and suggests that early interventions focusing on pain-related avoidance factors may have beneficial effects on the long-term outcomes in RA. Neither of the 2 previous meta-analyses addressed the effect of moxibustion on pain in patients with RA? Both of the reviews have not discussed the pain behaviors in their articles. The purpose of this systematic review and meta-analysis was to assess the effect of moxibustion on pain in patients with RA.

## 2 Methods

This meta-analysis was conducted according to the recommendations of the Preferred Reporting Items for Systematic Review and Meta-Analyses (PRISMA). As all the data collected and analyzed in this study were anonymous and previously published, and no direct patient information was used; thus, there was no need for ethical approval or patient consent.

A systematic literature search of MEDLINE, EMBASE, and Cochrane Library databases, as well as 3 Chinese databases (Wan Fang Med Database, CNKI, CBM) up to November 2018 was performed independently by 2 investigators (BS and QS). If consensus could not be reached, the third reviewer (GX) was consulted for a final decision. English-language articles for combinations of the following terms: “rheumatoid arthritis,” “RA,” and “moxibustion,” “moxa” were used. There were no language biases. Finally, review articles were searched, and lists of selected articles were screened and checked for 6 potential studies.

### 2.1 Types of studies

Prospective RCTs were included in this systematic review. We excluded trials in which moxibustion was part of a complex intervention as well as case studies, case series, qualitative studies, reviews, animal studies, and uncontrolled trials.

### 2.2 Types of intervention

We included studies that used any type of moxibustion (direct or indirect) for treating RA in any of the peripheral joints. Studies were included if moxibustion was used as the sole intervention or as an adjunct therapy in conjunction with another standard treatment for RA. Some other complex interventions were excluded, such as acupuncture, Chinese herbal medicine, or Chinese patent medicine.

### 2.3 Types of outcome measures

The outcomes were pain intensity ratings, measured with a 10-cm visual analog scale (VAS) anchored at each end with the phrases “no pain” and “worst pain imaginable” during the testing process. Inflammatory factors, such as erythrocyte sedimentation rate (ESR), C-reactive protein (CRP), and rheumatoid factor (RF), were also included. The response to treatment with moxibustion was assessed using the American College of Rheumatology (ACR) outcome measure, the ACR50 rate. In addition, the total response rate, which is mostly based on Chinese guiding principles, was also used as an outcome in this review.

### 2.4 Data extraction and quality assessment

The data from these articles were validated and abstracted according to predefined criteria that included author information. Data were processed in accordance with the Cochrane Handbook.^[9]^ All articles were retrieved and assessed independently by 2 reviewers who extracted data, including the authors, publication date, sample size, control intervention regimens, and main outcomes.

Intervention effects were presented using risk ratios (RRs) and 95% confidence intervals (CIs) for dichotomous data and mean differences (MDs) and 95% CIs for continuous data. The *χ*^2^ and I^2^ tests were used to measure statistical heterogeneity. A fixed-effect model was used if I^**2**^ < 50% and *P* > 0.1; otherwise, a random-effect model was used in the presence of statistically significant heterogeneity. I^2^ values of 0% indicated no heterogeneity, 25% indicated low heterogeneity, 25% to 50% indicated moderate heterogeneity, and 50% indicated high heterogeneity.^[10]^ The risk-of-bias was assessed using the risk-of-bias tool from the Cochrane Handbook for Systematic Reviews.

The following characteristics of the article were assessed: random sequence generation, allocation concealment, blinding of outcome assessment, blinding of participants and personnel, incomplete outcome data, selective reporting, and other biases. Our review used Low (L), Unclear (U), and High (H) categorizations. To assess the extent of publication bias, a funnel plot could be used. The meta-analysis was conducted with Rev-Man (version 5.3; Cochrane Collaboration, Copenhagen, Denmark).

## 3 Results

### 3.1 Studies included in the meta-analysis

As reported in PRISMA Flow Diagram, 184 of the 551 retrieved studies were excluded because of duplication. Three-hundred five studies were excluded based on the title and abstract, and 56 full-text articles were excluded because of the use of herbal medicine, different moxibustion comparisons, complex intervention, lack of data, or the small sample size. Finally, 6 studies comprising 281 patients were included in the meta-analysis.^[11–16]^

### 3.2 Main results and sensitivity analysis

**Figure 1:**
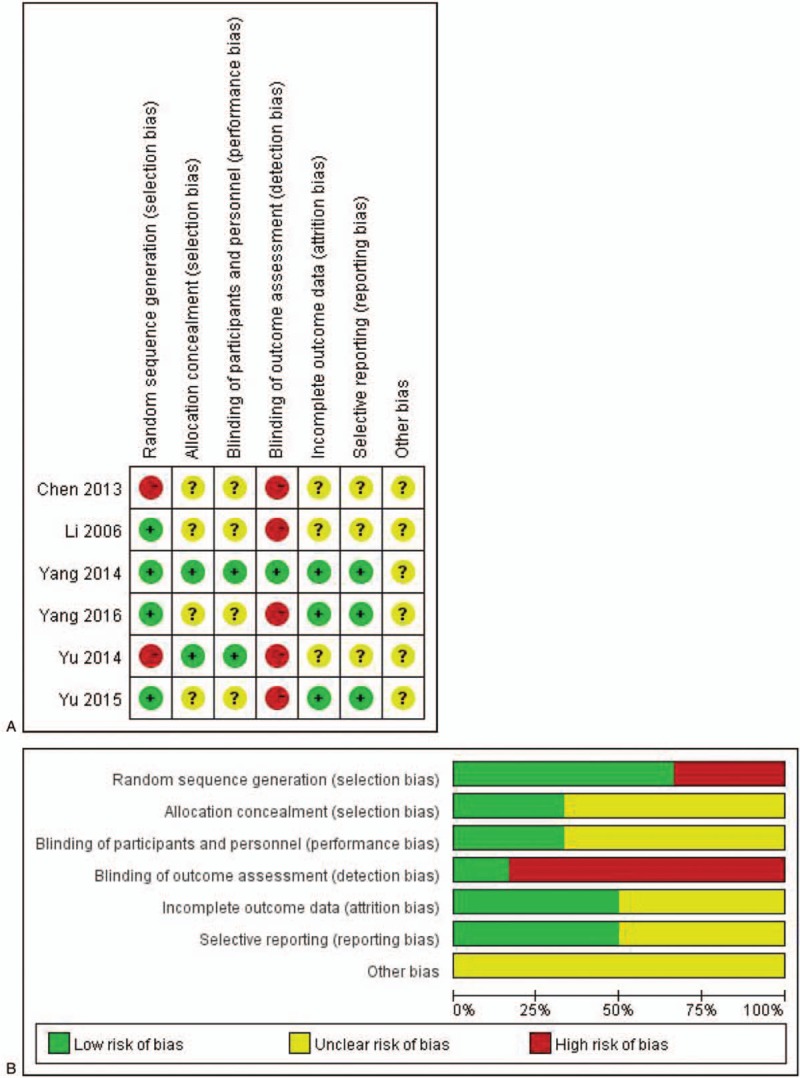
(A) Risk of bias graph: review authors’ judgments about each risk of bias item presented as percentages across all included studies. (B) Risk of bias summary: review authors’ judgements about each risk of bias item for each included study.

The characteristics of the included studies are presented in Table 1. The Cochrane risk-of-bias is presented in Fig. 1A and B.

**Table 1:**
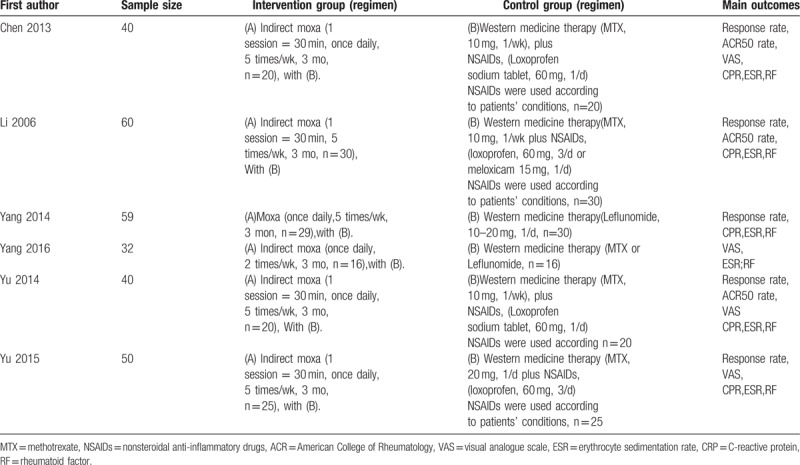
Summary of the randomized controls trials of moxibustion for rheumatoid arthritis (RA).

### 3.3 Pain on VAS

**Figure 2:**
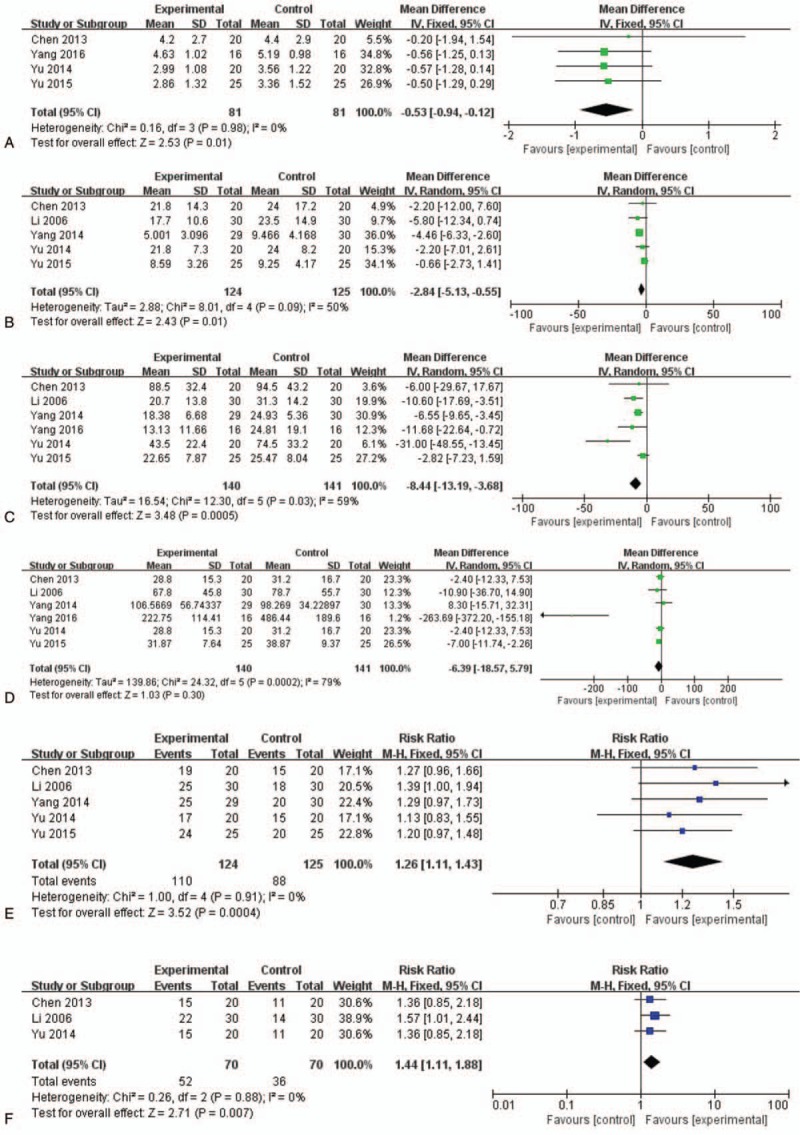
Moxibustion with western medicine therapy vs Western medicine therapy on (A) pain (VAS), (B) CRP, (C) ESR, (D) RF, (E) response rate, (F) ACR50.

Four RCTs^[11,14–16]^ reported pain (n = 162); a fixed-effects model was used because the heterogeneity test showed an I^2^ = 0% among the studies (*P* =.98). Meta-analysis indicated that, compared with Western medicine therapy, moxibustion did not significantly alleviate pain [MD = −0.53, 95% CI (−0.94, −0.12), *P* =.01] (Fig. 2A). The present study demonstrated that moxibustion might be effective for symptom management in patients with RA.

### 3.4 CRP

Five^[11–13,15,16]^ studies reported CRP for 249 participants. A random-effects model was used because the heterogeneity test showed I^2^ = 50% among the studies (*P* =.09). The results of the meta-analysis indicated that, compared with Western medicine therapy alone, moxibustion combined with Western medicine therapy had effects on CRP levels, with moderate heterogeneity among studies (MD = −2.84, 95% CI [−5.13, −0.55], *P* =.01) (Fig. 2B).

### 3.5 ESR

Six^[11–16]^ studies reported ESR for 281 participants. A random-effects model was used because the heterogeneity test showed I^2^ = 59% among the studies (*P* =.03). The results of the meta-analysis indicated that, compared with Western medicine therapy alone, moxibustion combined with Western medicine therapy had no significantly different effects on ESR, with high heterogeneity among studies (MD = −8.44, 95% CI [−13.19, −3.68], *P* =.0005) (Fig. 2C).

### 3.6 RF

Six^[11–16]^ studies reported RF involving 281 participants. A random-effects model was used because the heterogeneity test showed I^2^ = 79% among the studies (*P* =.0002). The results of the meta-analysis indicated that there was no difference between the effects of Western medicine therapy alone and moxibustion with Western medicine therapy on RF, with high heterogeneity among studies (MD = −6.39, 95% CI [−18.57, 5.79], *P* =.30) (Fig. 2D).

### 3.7 Response rate

Five RCTs ^[11–13,15,16]^ reported a response rate for moxibustion with Western medicine therapy compared with Western medicine therapy alone. A fixed-effects model was used because the heterogeneity test showed I^2^ = 0% among the studies (*P* =.91). All RCTs showed favorable effects of moxibustion on response rate. The meta-analysis also suggested that there were no significant differences between the 2 groups (n = 249, RR = 1.26, 95% CI (1.11, 1.43), *P* =.0004] (Fig. 2E).

### 3.8 ACR50 rate

Three RCTs^[11,12,15]^ (n = 140) reported on ACR50 as a measure of RA improvement. A fixed-effects model was used because the heterogeneity test showed I^2^ = 0% among the studies (*P* =.88). Two of the studies showed statistically significant positive effects on the ACR50 rate between groups, while the other did not. The results of meta-analysis suggested that, compared with Western medicine therapy alone, moxibustion with Western medicine therapy had statistically significant favorable effects on improving the ACR50 rate (n = 140, RR = 1.44, 95% CI [1.11, 1.88], *P* =.007) (Fig. 2F).

## 4 Discussion

Moxibustion treatments have been applied in RA in China for thousands of years. Our review aimed to add recent RCTs to update the evidence of the effects of moxibustion treatment in patients with RA. The result of this meta- analysis suggested potential benefits from moxibustion in alleviating pain, and increasing the response rate and ACR 50 rate. Meta-analysis of 4 studies showed significant reduction in pain, based on the therapeutic effective rate.

We also investigated the effect of moxibustion on inflammatory factors, such as CRP, ESR, and RF. While moxibustion treatments may have effects on CRP, the effects on ESR and RF were not significant. Both RA patients and their physicians often assume that pain is a result of inflammation,^[17]^ but there may be other mechanisms involved in pain, besides inflammation. Several studies have reported that the association between pain intensity and objective measures of inflammation is low.^[18,19]^ For example, Boyden et al^[19]^ reported a continuous spectrum of deficits in central nervous system pain processing mechanisms among individuals with RA. Moreover, a previous publication showed that autoantibodies to citrullinated proteins induce joint pain, independent of inflammation, via a chemokine-dependent mechanism.^[20]^ These studies all suggest that noninflammatory factors contribute to the expression of pain in RA. Therefore, the effect of moxibustion on inflammatory factors in RA should be interpreted with caution.

Compared with previous reviews, we identified new RCTs. The results of our review are similar to those reported in the previous 2 reviews.^[6,7]^ There are several factors for this outcome, such as the small number of studies, the type of studies included, the measures used across the studies, and limitations due to insufficient data, which may limit the power of our calculations.

Moxibustion is not only a treatment approach for RA, but is also an important component of TCM. At the same time, it is also a convenient approach in clinical practice. According to the TCM theory, moxibustion warms the interior and dissipates the cold, regulates and resolves stasis, softens and dissolves mass, resuscitates yang, and warms and activates the meridians.^[21]^ Previous studies have indicated that moxibustion could relieve chronic visceral hyperalgesia by activating the spinal dynorphin and orphanin-FQ system, decreasing hypothalamic corticotrophin-releasing hormone levels, and decreasing prokineticin-1 and prokineticin receptor-1 expression.^[22]^ Moxibustion could also enhance the pain threshold and restore sensitivity by decreasing 5-hydroxytryptamine concentration in the colon tissue.^[23]^ Although moxibustion has a positive effect on RA, the very elements of moxibustion that produce treatment effects also produce side-effects, such as burn wounds and pruritus when moxa is lit on or above the acupoints. In our study, One RCT^[13]^ reported the occurrence of burn wounds, pruritus, fatigue, blisters, and skin flushing in moxibustion. This suggests that there is a need to improve the quality control management in future studies. Moreover, in the pain management of RA, we should pay more attention to the complaints of patients’ pain, which provides a basis for our destined interventions.

However, the study had some limitations. First, the included studies involved small sample sizes, and they lack power calculations or adequate follow-up in controls. Second, the small number of studies, the type of studies, the measures used across the studies, and the limitations in terms of the provided data may limit the power of our calculations. Third, the fact that moxibustion interventions cannot be controlled limits the generalizability of the studies. All of the included trials were performed in China, which limited the generalizability of the research. Finally, we did not perform a publication bias test due to the insufficient number of eligible studies for each outcome. Thus, the robustness of our findings may be impaired by possible publication bias.

## 5 Conclusions

In this study, we found that moxibustion with Western medicine therapy is superior to Western medicine therapy alone for pain in patients with RA. Consequently, we concluded that moxibustion is an alternative for treating RA. Moxibustion had significant effects on pain in patients with RA, but the effects of moxibustion on inflammatory factors in RA were unclear. Further multicenter studies with larger sample sizes and high-quality RCTs are needed to verify our results.

## Author contributions

**Conceptualization:** Biyu Shen.

**Data curation:** Biyu Shen, Haoyang Chen, Yongchang Li, Xian Du.

**Formal analysis:** Qian Sun, Yongchang Li.

**Funding acquisition:** Biyu Shen.

**Methodology:** Biyu Shen, Qian Sun, Haoyang Chen, Xian Du.

**Resources:** Biyu Shen, Huiling Li.

**Software:** Biyu Shen, Qian Sun, Haoyang Chen, Yongchang Li, Xian Du.

**Supervision:** Huiling Li, Guang-yin Xu.

**Validation:** Huiling Li, Guang-yin Xu.

**Visualization:** Huiling Li, Guang-yin Xu.

**Writing – original draft:** Biyu Shen, Qian Sun, Haoyang Chen.

**Writing – review & editing:** Biyu Shen, Qian Sun, Haoyang Chen, Huiling Li, Guang-yin Xu.
